# Reduction of Human Mobility Matters during Early COVID-19 Outbreaks: Evidence from India, Japan and China

**DOI:** 10.3390/ijerph18062826

**Published:** 2021-03-10

**Authors:** Zhehao Ren, Ruiyun Li, Tao Zhang, Bin Chen, Che Wang, Miao Li, Shuang Song, Yixiong Xiao, Bo Xu, Zhaoyang Liu, Chong Shen, Dabo Guan, Lin Hou, Ke Deng, Yuqi Bai, Peng Gong, Bing Xu

**Affiliations:** 1 Ministry of Education Key Laboratory for Earth System Modeling, Department of Earth System Science, Tsinghua University, Beijing 100084, China; rzh18@mails.tsinghua.edu.cn (Z.R.); cauzhangtao@126.com (T.Z.); lim20@mails.tsinghua.edu.cn (M.L.); xiaoyixiong@mail.tsinghua.edu.cn (Y.X.); xu-b15@mails.tsinghua.edu.cn (B.X.); guandabo@tsinghua.edu.cn (D.G.); yuqibai@tsinghua.edu.cn (Y.B.); 2 MRC Centre for Global Infectious Disease Analysis, Department of Infectious Disease Epidemiology, Faculty of Medicine, School of Public Health, Imperial College London, London W2 1PG, UK; ruiyun.li@ibv.uio.no; 3 Centre for Ecological and Evolutionary Synthesis (CEES), Department of Biosciences, University of Oslo, 0316 Oslo, Norway; 4 Division of Landscape Architecture, Department of Architecture, The University of Hong Kong, Hong Kong SAR, China; binley.chen@hku.hk; 5 Center for Statistical Science, Tsinghua University, Beijing 100084, China; wc18@mails.tsinghua.edu.cn (C.W.); song-s19@mails.tsinghua.edu.cn (S.S.); liucy17@mails.tsinghua.edu.cn (Z.L.); c-shen18@mails.tsinghua.edu.cn (C.S.); houl@tsinghua.edu.cn (L.H.); kdeng@tsinghua.edu.cn (K.D.); 6 Department of Industrial Engineering, Tsinghua University, Beijing 100084, China; 7 Tsinghua Urban Institute, Tsinghua University, Beijing 100084, China; 8 Center for Healthy Cities, Institute for China Sustainable Urbanization, Tsinghua University, Beijing 100084, China

**Keywords:** human mobility, reproductive number, imported coronavirus disease 2019 (COVID-19), hospital bed shortage, duration from COVID-19 onset to diagnosis confirmation

## Abstract

Mobility restrictions have been a heated topic during the global pandemic of coronavirus disease 2019 (COVID-19). However, multiple recent findings have verified its importance in blocking virus spread. Evidence on the association between mobility, cases imported from abroad and local medical resource supplies is limited. To reveal the association, this study quantified the importance of inter- and intra-country mobility in containing virus spread and avoiding hospitalizations during early stages of COVID-19 outbreaks in India, Japan, and China. We calculated the time-varying reproductive number (*R*_t_) and duration from illness onset to diagnosis confirmation (*D*_oc_), to represent conditions of virus spread and hospital bed shortages, respectively. Results showed that inter-country mobility fluctuation could explain 80%, 35%, and 12% of the variance in imported cases and could prevent 20 million, 5 million, and 40 million imported cases in India, Japan and China, respectively. The critical time for screening and monitoring of imported cases is 2 weeks at minimum and 4 weeks at maximum, according to the time when the Pearson’s Rs between *R*_t_ and imported cases reaches a peak (>0.8). We also found that if local transmission is initiated, a 1% increase in intra-country mobility would result in 1430 (±501), 109 (±181), and 10 (±1) additional bed shortages, as estimated using the *D*_oc_ in India, Japan, and China, respectively. Our findings provide vital reference for governments to tailor their pre-vaccination policies regarding mobility, especially during future epidemic waves of COVID-19 or similar severe epidemic outbreaks.

## 1. Introduction

The novel coronavirus SARS-CoV-2 has appeared and affected human societies for more than a year as of this writing [1]. The outbreak was declared as public health emergency of international concern by the World Health Organization on 30 January 2020 [2]. With limited available vaccines, much of the effort made has been to suppress rapid diffusion of the disease by implementing non-pharmaceutical interventions such as restricting human movements [3]. Other non-pharmaceutical interventions include wearing face masks, proper hand hygiene, or changing climate or weather conditions [4,5]. Among these, controlling inter- and intra-country mobility is a top-down action that can be more correctly and uniformly implemented than enforcement of mask wearing and similar measures, and is much more controllable than altering local climate or weather. Mobility restriction can play an important role in protecting individuals from potential infection by interrupting disease transmission, until safe and effective vaccines can be made widely available [6]. In the present study, we therefore examined mobility restriction as a determinant of disease transmission.

Pilot studies have demonstrated the effect of both inter- and intra-country mobility restrictions on slowing the spread of COVID-19. For example, strict measures implemented in Wuhan, such as the cordon sanitaire, has impeded 80% of cases that could have been exported to other cities internationally [7]. Outside Wuhan, limiting human mobility, together with providing sufficient medical resources such as testing kits and hospital beds at an early stage has been a proven approach in China [8], Italy [9] and the United States (US) [10]. Moreover, strict and prompt restrictions on mobility have effectively protected local economies by limiting the spread of disease [11] and are cost-effective in the long run [12]. As for economic concerns, transportation was needed to sustain international supply-demand chains of resources [13]. However, human mobility is closely related to the number of confirmed cases of COVID-19 infection that may be owing to importation by inbound airline passengers [14,15,16,17,18]. Although case importation is the key factor driving the onset of local outbreaks, inter-country mobility cannot easily be unilaterally modified by a single country; therefore, imported cases cannot be completely prevented as it is impossible to completely isolate all countries in the world [19]. Therefore, understanding the effect of inter-country mobility on local transmission and number of cases with local ongoing epidemic is of great importance. There have been much early studies focusing on the effect of travel restrictions or airport screening with other interventions via mathematical models [7,8,20]. However, few model findings have suggested a proper quarantine period [4]. Determining the approximate period during which imported cases can spark local outbreaks is critical, to better control travelers’ mobility and impede local transmission in a timely manner. Apart from modeling, empirical studies are needed in different countries, to provide concrete data and develop tailored local policy.

Containing local transmission that is triggered by imported cases of COVID-19 requires greater attention [7,17,21]; for this, deeper insight into the effects of intra-country mobility on disease transmission is required. Taking provinces or states as the basic study area, it is apparent that moving from one to another can result in local, within-country case importation, which can lead to the disease spreading across an entire country. Owing to a lack of alert systems, China experienced this phenomenon through the end of 2019, and faced a critical shortage of medical resources in early February, 2020 [1,17,22], which sharply raised the crude fatality rate. To this end, reducing intra-country mobility via various measures, such as implementing social distancing policies to cut off virus transmission, can help to lower the number of infections and save needed medical resources. Although there has been much effort made worldwide in terms of early preparedness before the emergence of COVID-19 [23,24], shortages of hospital resources have still occurred in many countries, which can partly be attributed to the broken supply-demand chains. Since the beginning of the pandemic, much effort has been devoted to planning for resource sharing [25], framing of better systems to decide turn-over time [26], and most importantly, ensuring that sufficient beds are available for patients who require hospitalization [27]. However, how intra-country mobility dynamics affect medical resources, in particular hospital beds, remains unclear owing to a lack of precise data. When investigating the impact of intra-country mobility on an entire country, studies have focused on the duration from the date of illness onset to the date of a confirmed COVID-19 diagnosis (*D*_oc_). However, providing information of differences in *D*_oc_ among different locations has been neglected. In the present study, we proposed a crude model to estimate daily bed shortages, to clarify the effect of intra-country mobility restrictions.

In this study, we aimed to quantify the importance of both inter- and intra-country mobility in containing virus spread and conserving hospital bed availability during early outbreak periods, and to provide policy recommendations in cases of recurring outbreaks. Three countries with differing socioeconomic and epidemiological characteristics were selected. India has the second largest population and number of COVID-19 cases worldwide but has been highly understudied. Japan initially succeeded in suppressing the daily increment in cases but was later forced to implement another nationwide lockdown. China was the first country to achieve successful control of SARS-CoV-2 transmission, where the D_oc_ gradually declined to zero during the first 2 months of the pandemic; however, the country experienced a crisis in disease reoccurrence in March owing to imported cases. Therefore, we investigated the status of virus transmission in these three countries by estimating the time-varying reproductive number (*R*_t_) and *D*_oc_ via the integration of multiple data streams. The epidemic experience in China was divided into two phases in this study, to enable fair comparisons with India and Japan: (i) 1 January to 29 February, the discovery and recovery phases; (ii) 1 March to 16 June 2020, the period during which transmission was appropriately managed. The first phase was used to measure the effect of intra-country mobility on hospital bed shortages; the second phase was used to study the impact of inter-country mobility on disease transmission. The empirical critical period to effectively limit the impact of case importation was subsequently quantified. Finally, we put forth policy recommendations based on our results. This study directly and quantitatively bridges mobility and imported cases, local transmission, and hospital bed supply. These findings can provide useful information for the public regarding how reducing mobility can directly reduce local transmission and relieve the disease burden on the healthcare system. It is of the utmost importance to involve the government, to improve preparedness for the coming wave of COVID-19 or other outbreaks that constitute a public health emergency.

## 2. Materials and Methods

### 2.1. Data Sources

The mobility dataset consisted of both inter- and intra-country human movements. To capture inter-country mobility, the number of airline passengers between 231 regions worldwide were retrieved from the International Air Transport Association [28], covering 4453 airports from January 2019 to March 2020. Here, airflow data in March 2020 were regressed with imported COVID-19 cases. The number of potentially averted cases of imported infection in 2020 was then calculated by substituting annual summed airflow data in 2019 for that in March 2020 in the regression function.

Human mobility data for China, Japan and India were collected from two major datasets. More specific, real-time location records in China were obtained from smart phone users of Baidu’s location-based service apps which are released on Baidu Migration big data website (https://qianxi.baidu.com/ (accessed on 16 June 2020). Mobility data in India and Japan were collected from the Google Community Mobility dataset (https://www.google.com/covid19/mobility/ (accessed on 26 April 2020). In this study, we collected data in the same temporal period of confirmed cases and intra-country mobility for each country. Note that these data were collected at country level as the study sites were country-based.

Epidemiology data in three countries were collected from publicly available patient databases, listed in Table 1. In these databases, desensitized patient information was recorded separately, including symptom onset date, diagnosis confirmation date, removal date (either recovered or died), and source country (imported cases only). Based on these records, the *D*_oc_ of each patient was calculated as the duration from symptom onset to case confirmation, with further estimation of the time spent waiting for testing kits or an available hospital bed. To detect the effect of mobility during early outbreaks, we selected these periods in the three countries.

### 2.2. Estimation of Time-Varying Reproductive Number, R_t_

The basic reproductive number, is defined as the average number of new secondary infections caused by an infected individual during the infectious period in a fully susceptible population, and characterizes the transmissibility of a disease. If the value of *R*_t_ is greater than 1, the disease will continue to spread. Given the variation in transmissibility with interventions, we estimated the daily reproductive number *R*_t_ using the *EpiEstim* package in R. The main estimation process was as follows.

Serial interval is defined as the difference in symptom onset dates between secondary new infectees and their origin case, which is vital in estimating R_t_. Digesting observed daily new cases *I(t)*, and fitted serial interval *w_j_* using the Bayesian framework with a Gamma (a, b) prior distribution for *R*(*T*) for each time window T = [t1, t2], the Poisson likelihood-based method outputs the posterior distribution of *R(T)* as in Equation (1). Here we set *a* = 1, *b* = 5, and specified the serial interval to be the Gamma distribution with a mean of 7.5 days and a standard deviation of 3.4 days:

(1) $$R\left(T\right)|I,w∼Gamma\left(a+\sum_{t=t_{1}}^{t_{2}}I\left(t\right),\frac{1}{\frac{1}{b}+\sum_{t=t_{1}}^{t_{2}}\sum_{j=1}^{t}I\left(t-j\right)w_{j}}\right),$$

Scanning through *I(t)* with a sliding window of size *d*, i.e., *W_t,d_* = [t − d + 1, t], a time-varying estimate $\hat{R}\left(t\right)=\hat{R}\left(W_{t,d}\right)$ can be obtained for each day. That is to say, the time-varying reproductive number *R*_t_ equals the posterior mean of Equation (1), as in Equation (2):

(2) $$\hat{R}\left(t\right)=\frac{a+\sum_{i=t-d+1}^{t}I\left(i\right)}{\frac{1}{b}\;+\;\sum_{i=t-d+1}^{t}\sum_{j=1}^{i}I\left(i-j\right)w_{j}},\;t\leq T.$$

Note that we basically adopted Thompson’s method [29], except for the serial interval (SI) estimation. Because there is no available information on pairs of infectees and their origins, SI can only be set as constant [30]. Here we adopted *d* = 7. Imported cases were considered separately in this study, to better estimate the *R*_t_.

### 2.3. Estimation of D_oc_ and Epidemic Curves by Illness Onset Date

The duration from date of illness onset to case confirmation, *D*_oc_, represents the dynamic period of waiting for medical resources like testing kits or available hospital beds. *D*_oc_ can be calculated using deidentified data in the patient records in open data sources (Table 1), which contain information such as illness onset date, confirmation date, and confirmation location, as in Equation (3):

(3) $$D_{oc}^{p}=T_{c}^{p}-T_{o}^{p},$$

where $D_{\mathrm{oc}}^{p}$ denotes the *D*_oc_ from the pth piece of patient records. $T_{c}^{p}$ and $T_{o}^{p}$ represent the dates of confirmation and illness onset in the *p*th piece of records, respectively. These records are also location-labelled, on the basis of which *D*_oc_ for each city on each day can be estimated. Taking advantage of this, epidemic curve according to illness onset date can also be estimated as follows.

The distribution of *D*_oc_ on date $T_{c}$ can be estimated, as in Equation (4):

(4) $$P\left(D_{oc}=j|T_{c}\right)=P\left(T_{c}-T_{o}=j|T_{c}\right)=\frac{n(T_{c}-T_{o}=j\;|\;T_{c})}{n\left(T_{c}\right)},\;T_{c}=1,\ldots ,T,$$

where $n\left(T_{c}\right)$ denotes the number of confirmed cases on date $T_{c}$, $n(T_{c}-T_{o}=j\;|\;T_{c})$ denotes the number of cases whose confirmation date was $T_{c}$ but whose onset date was $T_{o}=T_{c}-j$.

Applying the distribution to officially reported confirmed COVID-19 cases, the number of patients on onset date $T_{o}$, $N\left(T_{o}\right)$, can be calculated using the Bayes formula of total probability, as in Equation (5):

(5) $$N\left(T_{o}\right)=\sum_{T_{c}=T_{0}}^{T}N\left(T_{c}\right)\cdot P(D_{oc}=T_{c}-T_{o}|T_{c}),$$

where $N\left(T_{c}\right)$ denotes the number of officially reported confirmed cases on date $T_{c}.$ $N\left(T_{o}\right)$ is the estimated total number of new-onset cases on date $T_{o}$.

### 2.4. Estimation of Bed Shortage

Disease onset-to-confirmation delay could result from limited diagnostic capacity and limited healthcare resources such as a shortage of hospital beds. This lack of resources was one of a fundamental drivers in the very early stages of disease transmission, as was the situation in Hubei Province at the beginning of 2020 (excluding Wuhan City, hereafter Hubei for short), which is relaxed after the development of *Fangcang* [31]. We assumed that the onset-to-confirmation time delay in Hubei characterized the time spent waiting for testing kits whereas in other locations, this is referred to the period waiting for both testing kits and hospital beds. Taking *D*_oc_ dynamics in Hubei as a natural decreasing trend in locations first experiencing a COVID-19 outbreak, the total number of person-days spent waiting for a hospital bed in unprepared locations can be estimated using the number of patients on the onset date, multiplied by the difference in *D*_oc_ between these locations and Hubei, summed over each onset day, as in Equation (6):

(6) $$N_{w}=\frac{\sum_{t=D_{1}}^{D_{T}}N^{UN}\left(t\right)\cdot \left(D_{oc}^{UN}\left(t\right)-D_{oc}^{HB}\left(t\right)\right)}{D_{h}},$$

where *N*_w_ denotes the total number of patients waiting for a hospital bed; $N^{UN}\left(t\right)$ denotes the number of patients in unprepared locations on the onset date *t*; $D_{oc}^{UN}\left(t\right)$ and $D_{oc}^{HB}\left(t\right)$ represent *D*_oc_ in unprepared locations and Hubei on illness onset date *t*, respectively; *D*_h_ is the average number of days patients are hospitalized; and $D_{1}$ and $D_{T}$ denote the start and end dates of the period with bed shortages, respectively.

### 2.5. Critical Period for Imported Case Prevention

Different periods were selected to calculate the Pearson’s correlation between imported cases and the daily reproductive number, which is defined from different start days to the end of the study period. The start date ranges from the first date with a confirmed case(s), to day 14 after that date. In correlation analysis, we took 1 week as the unit of calculation; the start week is defined as the first week following the start date. Thereafter, the correlations were calculated recursively from the first start week to the end week in 1-day steps. The empirical critical timing is when the correlations drop sharply. The entire study periods in India, Japan, and China are given in Table 1.

## 3. Results

### 3.1. Time-Varying Reproductive Number R_t_ and D_oc_

**Figure 1:**
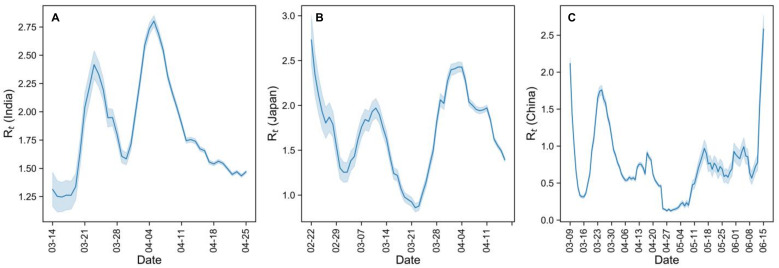
Time-varying reproductive number *R*_t_ in (**A**) India, (**B**) Japan and (**C**) China using R package, *EpiEstim*; parameter settings are described in Section 2.2. Note that the *R*_t_ in China was generally less than 1 despite ongoing virus spread.

The time-varying reproductive number *R*_t_ was estimated during the period when early outbreaks occurred (Figure 1). Across countries there was a large *R*_t_ at the beginning of the pandemic, indicating high transmissibility of COVID-19. Effective measures were put in place to reduce virus spread, which is reflected in the fluctuating *R*_t_ curves. Consistent with official declarations, Japan and China both succeeded in controlling virus transmission in late March (*R*_t_ < 1). However, Japan failed to maintain this trend, which resulted in a rebound. China was able to maintain virus control, until a local outbreak occurred in Beijing in June 2020. In contrast, we detected a high transmission rate in India at the beginning of the epidemic in that country, followed by a considerable reduction in late April. However, the *R*_t_ was consistently greater than 1, indicating that the disease continued to spread during the entire study period, only with different intensities.

**Figure 2:**
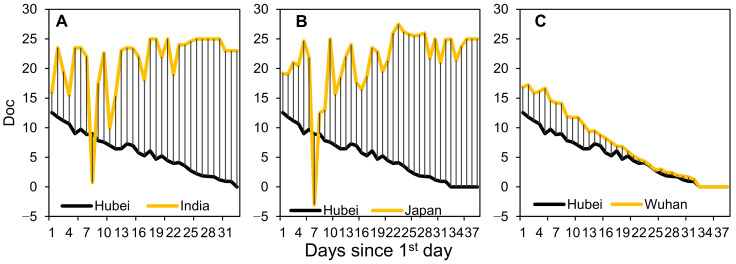
Dynamic *D*_oc_ and sketch of indents. *D*_oc_ in (**A**) India and Hubei, (**B**) Japan and Hubei, and (**C**) Wuhan and Hubei. Striped areas represent the difference in *D*_oc_ and can be used to roughly estimate bed shortages within a certain period.

The value of *R*_t_ reflects the rate of virus transmission, and *D*_oc_ indicates preparedness in terms of both medical resources and public awareness. Both India and Japan managed to reduce *D*_oc_ within approximately 7 days, which then soared to higher than the initial levels (Figure 2A,B). The *D*_oc_ during the second phase of epidemic in China was close to zero, with the severe and strictly enforced measures such as lockdown of entire cities during the first phase. Therefore, *D*_oc_ dynamics during the first phase, visualized in Figure 2C, gradually reduced from double digits to zero on 19 February 2020, indicating that wherever a symptomatic patient was identified, testing kits and a hospital bed were available.

### 3.2. Critical Period for Controlling Imported Cases

**Figure 3:**
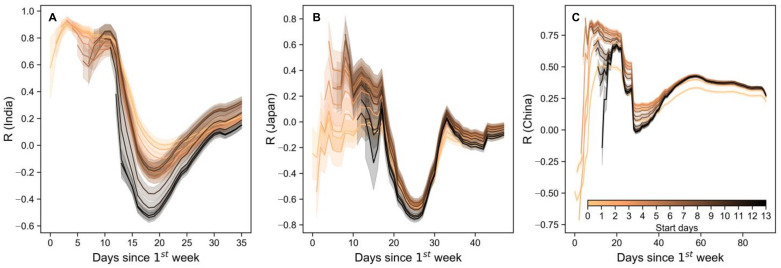
Pearson’s correlation between imported cases and time varying reproductive numbers in India (**A**), Japan (**B**), and China (**C**) with 14 temporal ranges. Start days here indicate the start day (since the first imported cases) of the temporal range.

The distinct correlation between transmissibility and number of imported cases (Figure 3) during the first 15-day period of documented local transmission indicated that controlling case importation is fundamental to modulating local outbreaks during the very early stages of the disease transmission. The strongest correlation occurred in the first 10 days (one start week with 3 subsequent days) in India, with peaks at 15 days in Japan, and 12 days in China. It is reasonable that these peak days denote the period with the greatest likelihood that imported cases can infect local people (exposed susceptibles), and is thus influenced by population density [32], or local interventions, such as social distancing or vaccination.

The drop day represents the date when local transmission is taken over by infected cases among local infectees, which reflects local surveillance capability for imported cases. The lowest correlation reached −0.6 in Indian and −0.8 in Japan, indicating that imported cases triggered local transmission, such that the driver of *R*t dynamics was no longer only imported cases. Note that the correlation drops sharply after 18, 24, and 26 days, in India, Japan and China, respectively. These days represent the time when the influence of imported cases on local transmission comes to an end, indicating the optimal time to subject imported cases to proper surveillance and monitoring.

### 3.3. Effect of Restriction of Inter-Country Mobility on Limiting Case Importation

**Figure 4:**
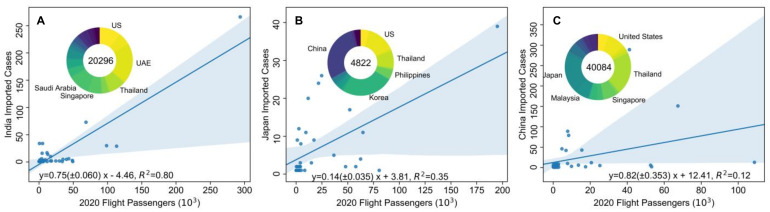
Imported cases in relation to inbound flight passengers in India (**A**), Japan (**B**) and China (**C**). Donut chart shows the estimated number of cases prevented by limiting inter-country mobility locally as compared with airline passengers in 2019. Values in the center of the chart indicate the possible number of confirmed imported cases that were prevented. Annotated countries are the top five greatest contributors to imported cases in each of the three countries. Data with country name are listed in Table A1.

A considerable reduction in inter-country mobility appeared to avert infections owing to imported cases. Our findings showed that the number of actual airline passengers in March 2020 with different originations can explain 80% and 35% of the variance in the corresponding imported cases in India and Japan, respectively (Figure 4). Statistical analysis indicated that every 10,000 passenger decrease in airline transport inflow resulted in a 7.5 (±0.6) and 1.4 (±0.35) decrease in imported cases in India and Japan, respectively (Figure 4A,B). Apparently, reducing the number of inbound passengers to 5950 alone could significantly diminish the linear impact of flight numbers on imported cases in India according to this linear regression results, although flight passengers less than 50,000 shows a trend towards the origin. Controls implemented on inbound terrestrial or oceanic passengers required greater restriction to control case importation in Japan. Moreover, regression analysis indicated that fewer flight passengers in 2020, as compared with the annual numbers in 2019, could have blocked an increase of more than 20 million imported cases in India (inset in Figure 4A), and approximately 5 million imported cases in Japan (inset in Figure 4B). China is a special case. Although limiting inter-country mobility is likely to have prevented importation of 40 million cases as shown in linear regression model (Figure 4C), inbound flight passengers only explain 12% of the variance of imported cases in China. This is discussed further in the following section.

Figure 4 (inserts) shows the countries with the greatest contributions to case importation in the three countries. We found that the United Arab Emirates, US, Saudi Arabia, Thailand, and Singapore were the five countries most responsible for imported cases in India. China, Korea, US, Thailand and the Philippines were the leading countries of case importation to Japan whereas Thailand, Japan, US, Singapore and Malaysia are the major countries responsible for imported cases in China.

### 3.4. Effect of Intra-Country Mobility on Hospital Bed Shortages

**Figure 5:**
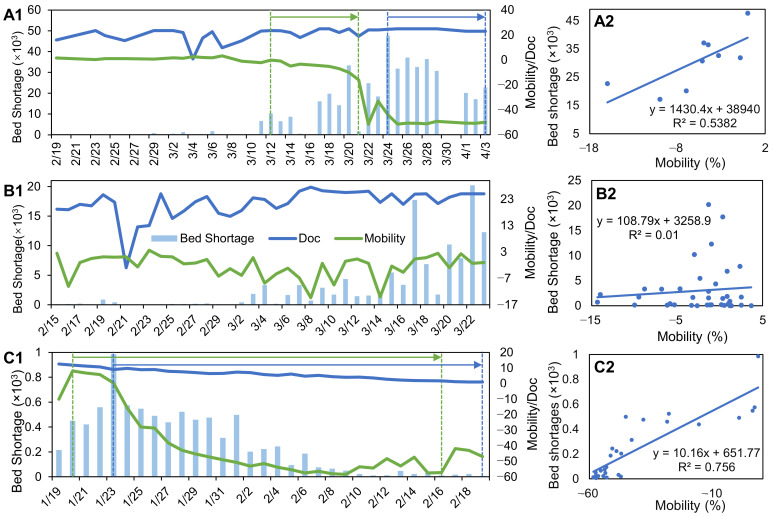
Dynamics of local *D*_oc_, intra-country mobility and hospital bed shortages in India (**A1**), Japan (**B1**) and China (**C1**). (**A2**), (**B2**) and (**C2**) denote the relationship between intra-country mobility and estimated bed shortages. Note that we consider the lagged relationship between the reduction of mobility and bed shortage by estimating the relationship in (**A2**) and (**C2**) using the mobility and bed shortage data in the period marked by green and blue arrows in (**A1**) and (**C1**).

Figure 5A,B show estimates of additional hospital beds in India and Japan. The estimates during the first phase in China are illustrated (Figure 5C) because no cases had been documented since the beginning of the second phase (Figure 2C). There was a continuous increase in bed shortages in India and China prior to the implementation of nationwide lockdown (24 March and 23 January, respectively) and a sharp decrease in intra-country mobility thereafter. Despite the similar increase of *D*_oc_ in Japan, measures such as a nationwide lockdown were still not implemented to reduce this. Judging from the curves and bars in Figure 5, there is a phase-wise relationship between intra-country mobility (curves in green) and bed shortages (bars). However, the bed shortage estimation is reasonable only after official actions such as lockdown have been taken.

Intra-country mobility and bed shortages were linearly regressed in India (since 24 March 2020), Japan and China (since 23 January 2020). Our results indicated that a 1% mobility release would result in 1430 (±501) bed shortages in India, 109 (±181) in Japan and 10 (±1) bed shortages in China. By integrating intra-country mobility and bed shortages, we demonstrate that the dynamics of intra-country mobility can explain 54%, 1%, and 76% of bed shortages in India, Japan, and China, respectively. The rate is extremely low in Japan because the precondition of beds shortage estimation was not fulfilled. This rate increases to 12% after the condition is plausibly met (Figure A2), which is further discussed in the following section.

## 4. Discussion

### 4.1. Mobility Restriction Versus Normal Condition

The initial high *R*_t_ in Figure 1 indicates an outbreak, which could have resulted from either imported cases or collective local infection; Figure 3 shows that the outbreak was owing to the former. This verifies the importance of monitoring and control measures for imported cases; empirically, their effect on local virus transmission peaked at approximately 2 weeks, was maintained at around 2 weeks, and ended at 4 weeks in all three countries. Similar to our Pearson’s correlation analysis, Sokadjo studied the relationship between inflow of airline passengers and daily confirmed cases [18]. Although we obtained a similar curve shape, we more directly calculated imported cases and reproductive numbers, yielding more robust data using recursive correlation analysis and more targeted results as we determined this relationship for each country. Our results do not repeat the work of Sokadjo’s but rather represent a clearer physical interpretation of a gap between the inflow of airline passengers in that previous study and our imported cases in the present work. Applying linear analysis between these two variables, imported cases proved to be the driver of initial local transmission as shown in Figure 3. Furthermore, in Figure 4, we illustrated that the greater the airline passenger inflow, the greater the number of imported cases, although with different intensities in different countries. Therefore, our sequential results re-assert the merit of exerting great effort to properly screen and manage all inbound flight passengers.

**Figure A1:**
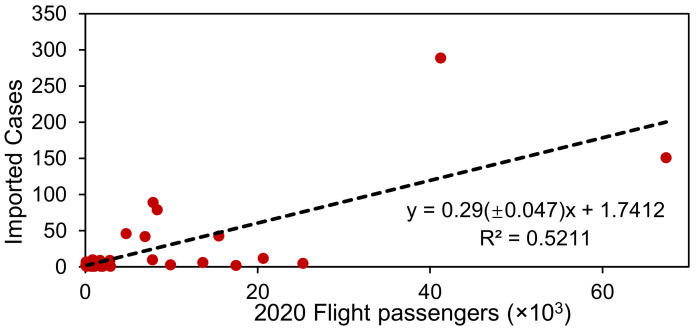
Imported cases in relation to number of inbound airline passengers in China, excluding low-risk countries namely, Thailand, Japan, and Cambodia.

Controlling flight inflow, however, does not necessarily mean stopping all inbound flights. First, according to the regressed results shown in Figure 4, there would still be imported cases in countries such as Japan and China, even if inter-country mobility were reduced to zero. Therefore, implementing the 15-day period to control imported cases, as in Figure 3, is of great importance to be able to restore normal daily life. During this period, greater preparedness is needed to ensure the availability of resources such as nucleic acid testing kits, hospital beds, and ventilators [23]. Second, as illustrated in the case of China, imported cases can be largely independent of airline travel, which can facilitate international travel as well as keeping the virus outside of the country. This “clean network” resulted from targeted policies for different flights [33]. Rather than using one-size-fits-all approach, China accepted most passengers but refused entry to those from countries with historically higher COVID-19 incidence rates, as a compromise. By excluding flights from low-risk countries such as Thailand, Japan and Cambodia, the explanation rate (R-square) would rise to 52% (Appendix A, Figure A1), which accords with the explanation rate of India and Japan. These evidences suggest two ways that a return to normal life can be achieved using inter-country mobility restriction.

Intra-country mobility adjustment also directly influences vital medical resources such as hospital beds. Once people became aware of the severity of COVID-19, tighter intra-country mobility policies led to fewer bed shortages (Figure 5A,C). Bed shortages were eliminated and patients could be attended as long as the difference between country-wide *D*_oc_ and the baseline had dropped to zero. Although total intra-country mobility restrictions can then be lifted, as happened during the second phase in China, this recovery is not totally equivalent to a return to normal life. Schlosser et al. found that suppressing intra-country mobility not only changes the intensity of restrictions, but also the structure of mobility [34]. Given limited finer location-based mobility indices, spatiotemporal behavior modes and land-use data, much information is lacking regarding how to measure when life can return to normal.

### 4.2. Prerequisite of Beds Shortage Estimation

*D*_oc_ proved to be a critical value in modeling the effect of imported cases on virus transmission in Kraemer’s work (Figure S2 in [35]); however, its capability to represent the level of preparedness among local governments and populations was neglected. As Figure 2A,B shows, the initial dynamics of *D*_oc_ in India and Japan suggested that patients with imported infections were aware of their symptoms and the government in each country managed to have them tested and admitted to hospitals. Thereafter, because public awareness remained at the same level, the most likely reasons accounting for the variance are the differences in preparedness regarding testing kits and hospital beds. Based on news reports in China, the difference of *D*_oc_ between Hubei and Wuhan is the difference between time spent waiting for testing kits only, and time spent waiting for both testing kits and hospital beds. The larger *D*_oc_ difference in India and Japan and the gradually smaller *D*_oc_ difference in China are the results of government measures and human behaviors, and provide a basis for the estimation of hospital bed shortages. Based on this reasoning, the bed shortage estimation can be correctly made when high levels of public awareness have been reached and the curve dynamics only result from different time spent waiting for hospital beds and/or testing kits. This prerequisite accounts for why intra-country mobility only explains 1% of the variance in bed shortage in Japan, which is extremely low compared to India and China. This is related to the underlying assumption of bed shortage estimation. 

**Figure A2:**
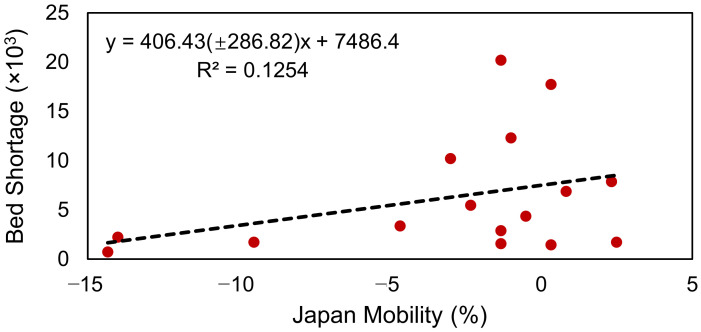
Intra-country mobility and hospital bed shortages in Japan since March 8 (the day with the lowest available data of intra-country mobility).

In Section 2.4, differences of two *D*_oc_ values are attributed to the period waiting for hospital beds only. However, *D*_oc_ could be high in Japan regardless of the testing kit and hospital bed availability if people fail to recognize the serious risks and are unwilling to be admitted to the hospital [36]. The general public in Japanese only became aware of the severity of the epidemic after a nationwide lockdown was initiated on 7 April [37], which is beyond the range of our data availability. Comparatively, the correlation reached 35% to 62% in India and China because the calculation begins from the date the cordon sanitaire was implemented. It can be reasonably concluded that only when people are alerted, do the differences of *D*_oc_ represent the time waiting for hospital beds. To verify this assumption, we took the day with lowest intra-country mobility as the date when the Japanese public became aware of the seriousness of the epidemic (March 8), and re-applied the regression analysis; the rate increased to 12.5% (Figure A2). This bed shortage estimation method is applicable only when the prerequisite is fulfilled.

### 4.3. Strengths, Limitations and Future Directions

This study succeeded in explaining the importance of both inter- and intra-country mobility using the aforementioned procedure. Most notably, estimation of hospital beds shortage is proposed based on *D*_oc_, which can be extended to all countries if its prerequisite is fulfilled. This contribution managed to provide medical resources data and, for the first time, provides the opportunity to directly correlate mobility with medical resources using only publicly available data. The importance of mobility during the early stages of COVID-19 outbreaks is thus highlighted.

Apart from aforementioned caveats, some limitations or uncertainties remain. First, the bed shortage estimation takes the *D*_oc_ of Hubei as baseline. However, because the outbreaks in India and Japan were later than that in Hubei, those countries may have had greater levels of preparedness regarding hospital beds and testing kits, whose actual *D*_oc_ baseline should be even lower than that of Hubei, resulting in underestimation of their bed shortage. This implies that, in reality, bed shortages may be even more sensitive to intra-country mobility, requiring a greater commitment from governments to restricting intra-country mobility. Second, the preciseness of *R*_t_ estimated using *EpiEstim* in R can be improved if information for pairs of infectees and their origin cases can be obtained.

Our quantitative analysis and plausible findings reinforce the importance of inter- and intra-country mobility regarding imported cases and medical resources, and explained the general process of case importation from other countries, proper screening and surveillance, local transmission, and hospital bed shortages. Future work should focus on developing a finer model and precisely distinguishing drivers of imported COVID-19 cases and hospital bed shortages. Further investigation on how daily human life can return to normal apart from adjusting the mobility level and structure, should be conducted to allow targeted policies to be developed.

## 5. Conclusions

This study empirically and quantitatively relates both inter- and intra-country mobility to imported COVID-19 cases and hospital beds, highlighting the importance of mobility during early stages of the outbreak. We analyzed the relationship between inter-country mobility, imported cases and time-varying reproductive number, and found that inter-country mobility led to more imported cases, which initiated local transmission of COVID-19 whose correlation with *R*_t_ peaks at around 2 weeks and drops at around 4 weeks, providing a reference for the optimal surveillance period. With local transmission, we estimated hospital bed shortages using the different durations from illness onset to case confirmation (*D*_oc_) in different countries, and found that intra-country mobility can explain 54%, 1%, and 76% of the variance in hospital bed shortages in India, Japan and China, respectively. Note that targeted airline passenger screening was implemented in China and the *D*_oc_ declined to zero during the second phase in that country. Therefore, both inter- and intra-country mobility could be relaxed to a large extent, providing a successful example in recovery of mobility. Although there are some limitations, the findings of this study can help the public to better understand the effect of mitigating both inter- and intra-country human mobility on limiting the diffusion of COVID-19 infection, and can provide quantitative results for policy makers in the face of recurring COVID-19 outbreaks and other epidemics. Efforts are needed to determine how best human life can return to normal in the face of the COVID-19 pandemic.

Based on our findings regarding the transmission process during the early outbreak stages, we put forth the following targeted policy recommendations: (1) Targeted policies must be designed to determine the maximum number of inbound flights that can be allowed, considering the incidence rate in the originating country, as China is currently doing. A balance must be struck between inbound travel (most importantly, to maintain supply chains) and COVID-19 case importation to avoid halting all air transport; (2) Strict screening of inbound passengers and proper monitoring of travelers suspected of imported infection, for a minimum of 2 weeks and maximum of 4 weeks; (3) Intra-country mobility restrictions must be enforced to varying degrees, which can help raise public awareness, and directly help to relieve the burden on local medical resources in the face of local transmission; (4) Ensuring adequate supplied of medical resources in preparation for infectious disease outbreaks and reducing the *D*_oc_ to near zero and maintaining *R*_t_ values to less than 1. Only then, can intra-country mobility restrictions be lifted.

## Figures and Tables

**Table 1:** Publicly available patient records databases used in this study.

Data Source	Country	Duration
https://www.covid19india.org/	India	2 February 2020–26 April 2020
https://github.com/reustle/covid19japan-data/tree/master/docs/patient_data	Japan	15 January 2020–17 April 2020
https://github.com/beoutbreakprepared/nCoV2019	China	1 January 2020–16 June 2020
https://github.com/Juan-ZJ/COVID-19-line-list		

## Data Availability

The datasets analyzed in this study can be found in Table 1.

## Appendix

**Table A1:** Inbound Flight passengers and imported cases from different source countries to India, Japan, and China in 2020.

India			Japan			China		
Source Country	2020 Flight Passengers	Imported Cases	Source Country	2020 Flight Passengers	Imported Cases	Source Country	2020 Flight Passengers	Imported Cases
Australia	29,673	2	Australia	47,913	2	Angola	75	3
Bahamas	20	1	Austria	3227	1	Austria	892	3
Bahrain	16,867	2	Belgium	3020	2	Belgium	1695	5
Bangladesh	18,229	2	Bolivia	34	1	Brazil	1709	9
Brazil	1512	3	Brazil	4121	2	Burkina Faso	82	7
Canada	24,496	2	Canada	14,771	3	Cambodia	52,633	6
China	1403	4	China	51,970	17	Canada	20,638	12
Congo	162	1	Congo	0	1	Denmark	642	1
Denmark	2352	1	Cote d’Ivoire	0	1	Egypt	1818	1
Egypt	4438	1	Czech	1787	1	Ethiopia	2024	1
Finland	1118	1	Egypt	2622	12	France	8352	79
France	12,690	14	Ethiopia	252	2	Germany	13,638	6
Germany	16,993	10	Finland	3951	3	Greece	1097	1
Greece	943	1	France	24,718	26	Hungary	722	4
Guyana	14	1	Germany	17,248	9	Iceland	127	1
Indonesia	11,687	17	Holland	4512	8	Indonesia	9901	3
Iran	688	34	India	8327	1	Iran	2829	9
Ireland	3841	2	Indonesia	36,538	5	Ireland	940	5
Italy	5146	34	Ireland	1137	9	Italy	4736	46
Japan	7318	2	Italy	9411	11	Japan	53,170	2
Kenya	5844	1	Korea	80,459	1	Malaysia	25,231	5
Malaysia	33,847	1	Mexico	4849	1	Netherlands	2717	6
Mexico	508	1	Morocco	1858	1	Niger	60	2
Netherlands	5761	3	New Caledonia	2372	1	Nigeria	845	10
New Zealand	5714	1	Philippines	64,747	11	Norway	443	2
Oman	49,183	1	Portugal	1220	2	Pakistan	1444	8
Philippines	3900	6	Spain	11,619	20	Philippines	15,501	43
Portugal	1051	1	Switzerland	3045	2	Russian Federation	6923	42
Qatar	39,981	5	Thailand	62,414	4	Saudi Arabia	585	4
Russian Federation	11,748	1	UK	21,669	24	Serbia	1223	4
Saudi Arabia	98,599	30	US	194,938	39	Singapore	17,491	2
Singapore	48,595	3	Vietnam	57,775	2	Spain	7846	89
South Africa	5942	1				Sweden	906	1
Spain	4512	16				Switzerland	1522	8
Sri Lanka	34,801	5				Thailand	108,739	13
Sweden	1651	3				Turkey	1291	2
Switzerland	4050	2				UAE	7785	10
Thailand	43,419	6				UK	41,216	289
Trinidad	16	1				US	67,375	151
Turkey	9125	4				Vietnam	2923	1
UAE	293,587	266						
UK	68,504	73						
US	113,165	29						
